# Bending Strain and Bending Fatigue Lifetime of Flexible Metal Electrodes on Polymer Substrates

**DOI:** 10.3390/ma12152490

**Published:** 2019-08-06

**Authors:** Tae-Wook Kim, Jong-Sung Lee, Young-Cheon Kim, Young-Chang Joo, Byoung-Joon Kim

**Affiliations:** 1 Materials Research Centre for Energy and Clean Technology, School of Materials Science and Engineering, Andong National University, Andong 36729, Korea; 2 Department of Materials Science and Engineering, Seoul National University, Seoul 08826, Korea

**Keywords:** flexible, reliability, bending strain, metal electrode

## Abstract

As the technology of flexible electronics has remarkably advanced, the long-term reliability of flexible devices has attracted much attention, as it is an important factor for such devices in reaching real commercial viability. To guarantee the bending fatigue lifetime, the exact evaluation of bending strain and the change in electrical resistance is required. In this study, we investigated the bending strains of Cu thin films on flexible polyimide substrates with different thicknesses using monolayer and bilayer bending models and monitored the electrical resistance of the metal electrode during a bending fatigue test. For a thin metal electrode, the bending strain and fatigue lifetime were similar regardless of substrate thickness, but for a thick metal film, the fatigue lifetime was changed by different bending strains in the metal electrode according to substrate thickness. To obtain the exact bending strain distribution, we conducted a finite-element simulation and compared the bending strains of thin and thick metal structures. For thick metal electrodes, the real bending strain obtained from a bilayer model or simulation showed values much different from those from a simple monolayer model. This study can provide useful guidelines for developing highly reliable flexible electronics.

## 1. Introduction

**Figure 1:**
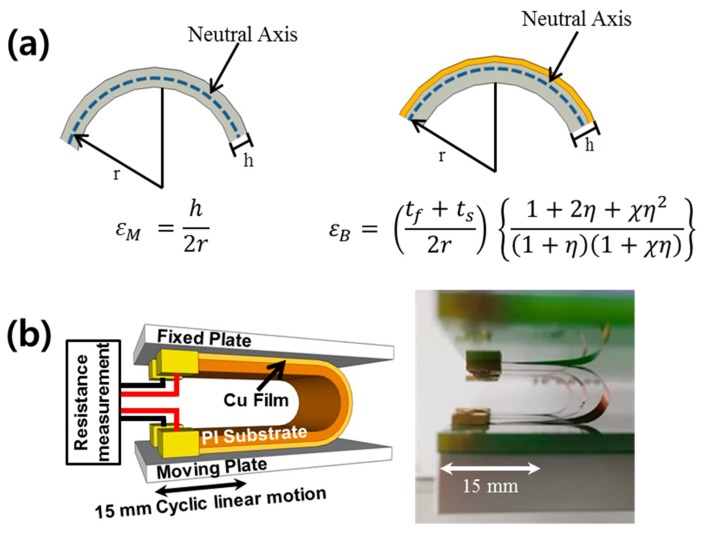
(**a**) Schematic illustrations and equations for calculating bending strain using monolayer and bilayer models. (**b**) Schematic and image of a bending fatigue test and an electrical resistance monitoring system.

Recently, electronic devices have been progressing towards flexible electronics, such as flexible displays, batteries, solar cells, and sensors [1], to increase performance and portability and to reduce weight. Flexible electronics are operated with repeated mechanical deformations, including bending, rolling, and twisting, so their mechanical reliability during repeated deformations is a critical hurdle to reaching real commercial viability [2,3,4]. When an external mechanical stress is applied to electronic devices, several mechanical reliability problems can occur in the metal layer, which is an essential part for electrical connections. Large deformations beyond a rupture strain fracture of the metal layer [5,6,7,8,9,10] and repeated small deformations even below a rupture strain can also cause mechanical and electrical failure because of metal film fatigue [11,12,13,14,15,16,17,18]. Therefore, an exact model of the applied mechanical stress during bending is necessary to design highly reliable flexible electronics. The most commonly used model to express bending strain is shown in Equation (1) [19]:

*ε*_M_ = *h*/2*r* (1)

where *ε*_M_ is the bending strain of a monolayer model, *r* is the bending radius, and *h* is the sample thickness. This equation is based on the curvature relationship and is derived from the strain evolution of monolayer materials when bending, as shown in Figure 1a. Generally, the metal layer is much thinner than the polymer substrate, so the equation assumes that a monolayer is applicable to measure bending strain.

To calculate the bending strain of multilayer electronics more exactly, we also proposed a bilayer model with a thin film on the substrate, as described in Equation (2) [20]:

*ε*_B_ = ((*t*_f_ + *t*_s_)/2*r*){(1 + 2*η* + *χη*^2^)/(1 + *η*)(1 + *χη*)} (2)

where *ε*_B_ is the bending strain of the bilayer model, *t*_f_ is the thickness of the metal film, *t*_s_ is the thickness of the substrate, *η* is the film thickness divided by the substrate thickness (*t*_f_/*t*_s_), and *χ* is the Young’s modulus of the film divided by the Young’s modulus of the substrate (*Ε*_f_/*Ε*_s_). According to the bilayer model, the mechanical stress evolution in a flexible metal electrode changes significantly depending on the sample structure: the thicknesses and mechanical properties of the metal film and polymer substrate [21,22]. Although the effect of the thickness of metal films on fatigue lifetime was reported in our previous paper, our main focus was only on how microstructure changes affected the metal film thickness [23]. The strain evolution of a multilayer structure and its effect on fatigue lifetime is still unclear. Furthermore, an in-depth study of the differences between a monolayer and a bilayer model of bending strain is necessary in order to clarify what is a reasonable calculation of bending strain. 

In this study, we investigated the bending strain and long-term fatigue lifetime of a metal electrode on a polymer substrate. We measured the electrical resistance of flexible metal electrodes in situ during repeated bending deformations. We compared and analyzed the mechanical reliability of different thicknesses of metal film and polymer substrate based on the bending strain obtained from monolayer and bilayer models for calculating bending strain. We used a finite-element method (FEM) simulation to determine the precise bending strain and compared it with those from analytic calculations. We analyzed the difference between the monolayer and bilayer strain calculations and discuss the proper method that depends on the sample structure. This study can provide helpful information to predict the exact mechanical reliability of flexible electronics.

## 2. Experimental 

We deposited Cu films with thicknesses of 100 nm and 1 µm on polyimide (PI/Kapton) substrates with thicknesses of 50, 75, and 125 µm by thermal evaporation at 1 × 10^−6^ Torr; the deposition rate was 50 nm/min. We performed the bending fatigue test by using a sliding bending tester (CKSI, Suwon, Korea), as shown in Figure 1b. Metal thin films were gripped by two grips at both edges and then set to a curved shape between plates. The upper plate was fixed, and only the lower plate had a repeated linear sliding motion that bent our samples [14,15,23,24]. This bending fatigue test is called a sliding-plate test in international standardization [25]. The sliding stroke was 15 mm, sliding frequency was 3 Hz, and the maximum bending cycle was 1 × 10^5^ cycles. We measured the electrical resistance in situ by a four-wire method (Keithley, 2700 Multimeter, Cleveland, OH, USA). We simultaneously tested at least three samples for statistical treatment. We controlled the bending radius by changing the gap of the two plates. We varied the bending radius from 1.65 to 8.9 mm, which corresponded to a bending strain of 0.7–1.5% calculated for the monolayer model. 

After the bending fatigue test, the surface of the metal film was observed by field-emission scanning electron microscopy (FESEM, MYRA3 XMH, TESCAN, Brno, Czech Republic). For comparison with the experimental results, we designed a FEM model with different thicknesses of the Cu film and PI substrate layer using a simulation program (ABAQUS, Dassault Systemes, Vélizy-Villacoublay, France). We analyzed four FEM models of different Cu and substrate thicknesses. For the FEM simulation, the Young’s modulus of Cu and PI was 128 and 2.5 GPa, and the Poisson’s ratio was 0.36 and 0.34, respectively [26,27].

## 3. Results and Discussions

**Figure 2:**
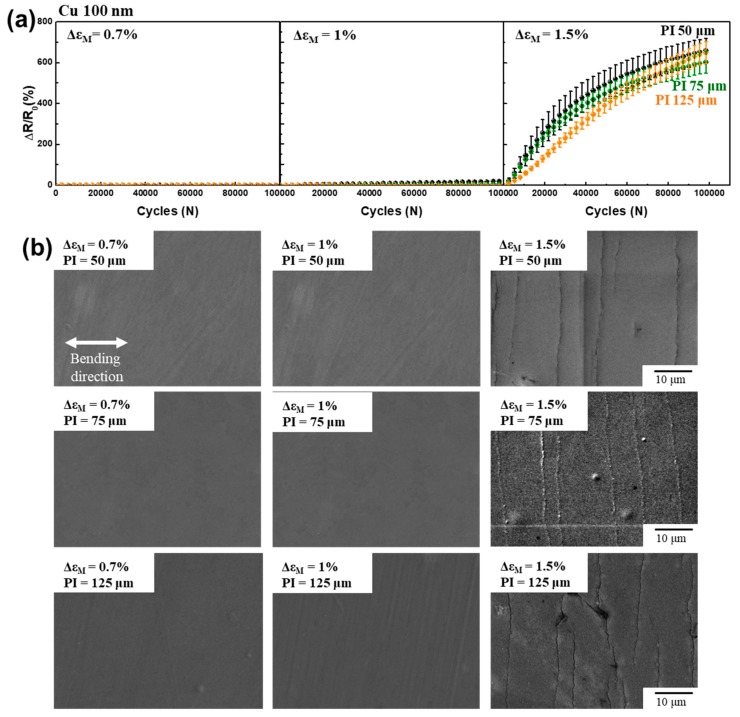
(**a**) Normalized electrical resistance changes of 100 nm thick Cu film on 50, 75, and 125 µm thick polyimide (PI) as a function of the number of bending cycles under bending strain amplitudes of 0.7%, 1%, and 1.5%. (**b**) SEM images of 100 nm thick Cu film after the bending fatigue test.

Figure 2a shows the changes in the electrical resistance of Cu with a thickness of 100 nm deposited on PI substrates with thicknesses of 50, 75, and 125 µm during the sliding-plate test under bending strain amplitudes of 0.7%, 1%, and 1.5%. We adjusted the bending radius to satisfy the target bending strain calculated for the monolayer bending strain (Δ*ε*_M_). For example, to test a 0.7% bending strain, the bending radii of samples with PIs that were 50, 75, and 125 µm thick were set at 7.14, 10.7, and 17.8 mm, respectively. The initial resistances of 100 nm thick Cu film on PI substrates with thicknesses of 50, 75, and 125 µm were 2.33, 2.24, and 2.43 Ω, respectively. The surface of the as-deposited metal film was very smooth and no cracks were detected before the fatigue test. For a 0.7% bending strain amplitude, we observed no change in electrical resistance until 1 × 10^5^ bending cycles in all three samples, and fatigue damage was not observed, as shown in Figure 2b. For the 1% bending strain amplitude, the electrical resistance change of the sample with a 50 μm thick PI after 1 × 10^5^ bending cycles was almost 0%. The sample with 75 μm thick PI and the sample with 125 μm thick PI exhibited slight resistance increases of 6% and 2%, respectively. We detected no fatigue damage in the metal film after the 1% fatigue test, as shown in Figure 2b. However, as the applied bending strain increased up to 1.5%, the electrical resistance increased significantly, as shown in Figure 2a. All the electrical resistances of the samples with 50, 75, and 125 μm thick PI substrates increased similarly, by about 600%. We observed fatigue cracks after the 1.5% bending fatigue test, as shown in Figure 2b. The cracks were straight and a few extrusions were observed, which is a typical brittle fatigue fracture of nanometer-scale metal films [16,23,26,28]. Because nanometer-scale metal films are strong and lack plasticity, brittle fractures occurred during the bending fatigue test [29,30].

**Figure 3:**
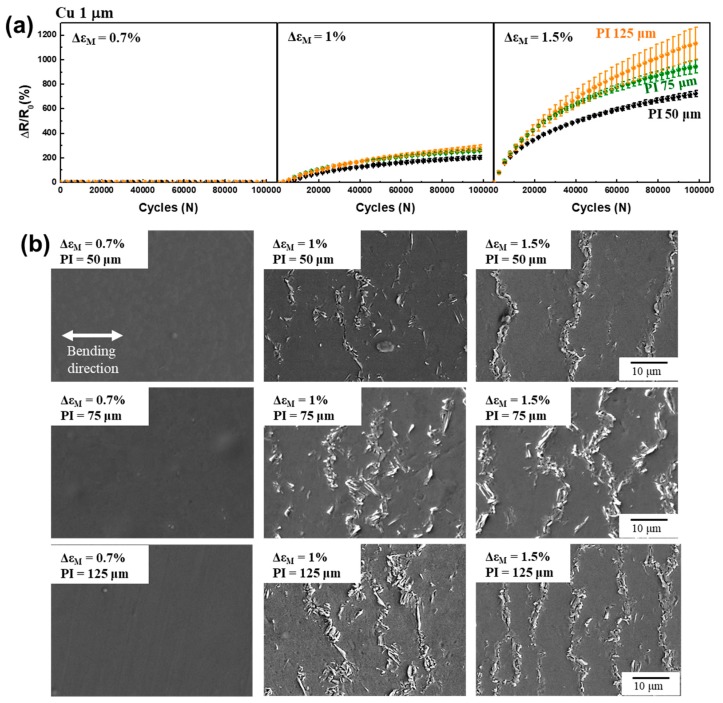
(**a**) Normalized electrical resistance changes of 1 μm thick Cu film on 50, 75, and 125 μm thick PI as a function of the number of bending cycles under bending strain amplitudes of 0.7%, 1%, and 1.5%. (**b**) SEM images of 1 μm thick Cu film after the bending fatigue test.

Figure 3a shows the electrical resistance changes of the 1 μm thick Cu film during the bending fatigue test under strain amplitudes of 0.7%, 1%, and 1.5%. The initial resistance of the 1 μm thick Cu film on PI substrates with thicknesses of 50, 75, and 125 µm was 0.18, 0.20, and 0.17 Ω, respectively. For the 0.7% bending strain amplitude, we observed no change in electrical resistance after 1 × 10^5^ cycles of all three specimens and did not observe fatigue damage after the bending fatigue test on the surface, as shown in Figure 3b. After 1 × 10^5^ cycles of repeated bending of 1% strain amplitude, the electrical resistance increased 207%, 261%, and 284% for the samples with 50, 75, and 125 μm thick PIs, respectively. As shown in Figure 3b, we observed extrusions and adjacent cracks on the surface of the 1 μm thick Cu film after the 1% bending fatigue test, which is a typical fatigue damage morphology of thick metal films [14,15,16,17,23,30]. During repeated bending deformations, irreversible dislocation movements in the metal film formed extrusions at the surface that acted as stress concentration sites. The cracks formed near the extrusions, propagated during further bending cycles, and finally resulted in increased electrical resistance [15]. 

Interestingly, for the 1.5% bending fatigue test, the Cu film on the thicker PI substrate showed a greater increase in electrical resistance. The resistance increases after 1 × 10^5^ cycles of a repeated bending test of 1.5% strain amplitude were 730%, 954%, and 1146% for the samples with 50, 75, and 125 μm thick PI, respectively. Furthermore, we detected more cracks on the surface of the 1 μm thick Cu film than on the thicker PI substrate, as is consistent with the greater increase of electrical resistance of thicker PI substrate. It should be noted that the 1 μm thick Cu film showed a different resistance change that depended on the PI substrate thickness in the 1.5% bending fatigue test (Figure 3a), but the 100 nm thick Cu film showed a similar resistance increase (Figure 2a). From the resistance change and crack morphologies, it can be argued that the effective mechanical stress significantly depends on the thickness of the metal film and the polymer substrate as well. 

**Figure 4:**
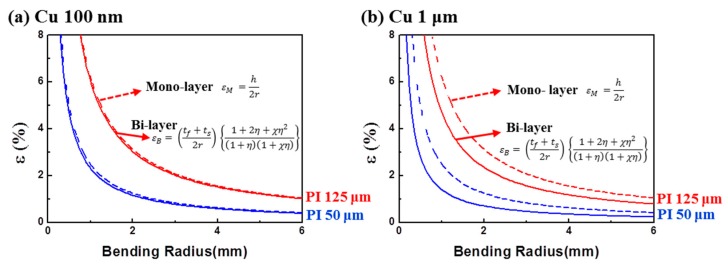
Calculated bending strains of (**a**) 100 nm thick Cu film and (**b**) 1 μm thick Cu film using monolayer and bilayer models.

To determine the exact bending strain of the metal film, we calculated the bending strains by using monolayer (Equation (1)) and bilayer (Equation (2)) models. Figure 4a shows the bending strains of 100 nm thick Cu films on the 50 and 125 μm thick PI. As the bending radius decreased, the bending strain of the metal film increased, and the metal film on the thinner polymer substrate showed less bending strain at a fixed bending radius because of a smaller mismatch from the neutral axis of the bending shape. Interestingly, for the 100 nm thick Cu film, the bending strains calculated from the monolayer and bilayer models were almost identical (Figure 4a), which implies that a thin metal film, such as the one that was a few hundred nanometers thick, does not cause the change of neutral axis and bending strain. In contrast, for the 1 μm thick Cu film (Figure 4b), the bending strains definitely differed with the strain calculation model: the bending strain obtained from the bilayer model was lower than that from the monolayer model. This implies that a thick metal layer, such as the one that was microns thick, may alter the stress distribution during bending deformation; the difference is quite large and cannot be negligible. Therefore, the bending strain should be carefully considered for thick metal films.

**Figure 5:**
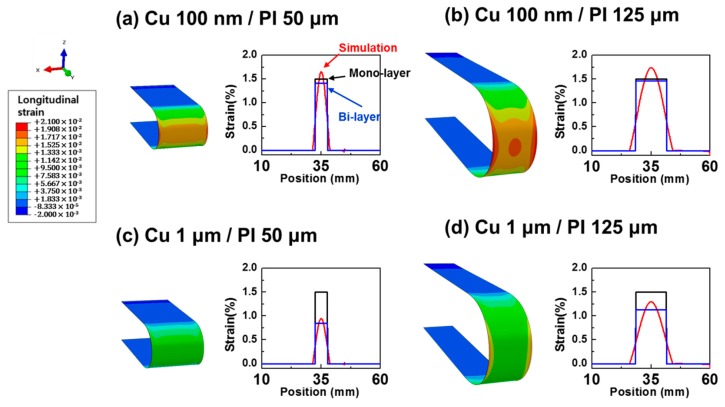
Finite-element method (FEM) modeling and strain distributions: (**a**) 100 nm thick Cu on 50 μm thick PI, (**b**) 100 nm thick Cu on 125 μm, (**c**) 1 μm thick Cu on 50 μm thick PI, and (**d**) 1 μm thick Cu on 125 μm thick PI.

To investigate the distribution of the actual bending strains of the metal films, we carried out a 3D FEM simulation, as shown in Figure 5. We applied two types of metal films (10 nm and 1 μm thick Cu) and two types of substrates (50 and 125 μm thick PI) to each model; so, four models were simulated. To make the bending shape, we placed rigid plates on each end of a sample and moved them toward each other until they reached the target bending radius condition. Figure 5 shows the FEM models for bending radii of 1.65 and 4.15 mm, which correspond to a 1.5% strain calculated for the monolayer model. Three kinds of bending strains as a function of position along the sample length are plotted in Figure 5: strains obtained from the monolayer, bilayer, and simulation models. In Figure 5a, the bending strains from the monolayer and bilayer models showed a rectangular shape, which was almost 1.5% at the bending area but 0% at the undeformed area because the equation for the monolayer or bilayer assumes that the bending curvature is identical along all sample areas. However, when the sample is bent in a U shape, the bending strain and curvature are changed continuously from the undeformed region to the bending region, so the bending strain from the simulation showed a continuous distribution, as shown in Figure 5a. 

For the models of the 100 nm Cu layer (Figure 5a,b), the bending strain from the simulation was similar to those from the monolayer and bilayer models because the thin metal film did not have a significant stress change. The maximum strain of the Cu surface changed slightly from 1.66% to 1.74% when the thickness of the PI substrate increased from 50 to 125 µm. That is consistent with the experimental result in Figure 2a, which showed that the increases of resistance were almost the same, even though the thickness of the polymer substrate was changed. In contrast, for the 1 μm Cu layer (Figure 5c,d), the bending strain from the simulation was much different from those from the monolayer and bilayer models because the thick metal film resulted in a stress distribution change, as discussed in Figure 4b. The maximum strain of the Cu surface increased from 0.95% to 1.30% when the thickness of the PI substrate increased from 50 to 125 µm. The difference in the strain was about 36%, which was enough to cause a difference in fatigue lifetime. This result matches well with the experimental data in Figure 3a, which showed a higher resistance change with a thicker PI substrate. In this study, we used the same evaporation process to exclude the adhesion effect and to focus on the effect of the thicknesses of the metal film and substrate. The fatigue lifetime can be changed by optimizing the adhesion between the metal film and the polymer substrate.

In summary, for the thin metal film, the bending strains obtained from the monolayer, bilayer, and simulation models were almost the same (Figure 5a,b), and the fatigue lifetime was also similar, regardless of PI thickness (Figure 2a). However, for the thick metal film, the bending strain from the simple monolayer model was much different from those from the bilayer and simulation models, and eventually, the fatigue lifetime differed according to the PI thickness change (Figure 3a). Anticipation of the bending strain is an important issue for designing highly reliable electronics. From our study, it can be argued that the bending strain of a flexible electrode having a thick metal layer should be considered carefully, and a bilayer model or FEM simulation is recommended for obtaining the exact bending strain.

## 4. Conclusions

We investigated the bending fatigue behavior of Cu film on PI substrate by in situ electrical resistance measurement. For the 100 nm Cu film, we observed a similar electrical resistance change. On the other hand, for the 1 μm thick Cu film, as the thickness of the PI substrate increased, we observed a greater electrical resistance increase and more cracks were formed. We calculated the bending strains of metal films using both monolayer and bilayer models and found that when the metal film was thick, the real bending strain from the bilayer model was much different from that from the monolayer model because the high elastic modulus of the metal film affected the total strain evolution during bending deformation. We also performed a FEM simulation to investigate the bending strain distribution. For the thin metal film, the bending strain from the simulation was similar to those from the monolayer and bilayer models because the thin metal film did not cause a significant stress change in the metal film. However, for the thick metal film, the bending strain from the simple monolayer differed greatly from those from the bilayer and simulation, and eventually, the fatigue lifetime differed according to the PI thickness change. In conclusion, when the metal electrode is thick, the bending strain needs to be exactly calculated using a bilayer model or simulation. This study provides useful information for developing highly reliable structures for flexible electronics.
